# Clinical and oncologic outcomes of posterior only total en bloc spondylectomy for spinal metastasis involving third lumbar vertebra: A case series

**DOI:** 10.1097/MD.0000000000037145

**Published:** 2023-02-02

**Authors:** Permsak Paholpak, Tadatsugu Morimoto, Taweechok Wisanuyotin, Winai Sirichativapee, Wilasinee Sirichativapee, Weerachai Kosuwon, Yuichi Kasai, Hideki Murakami

**Affiliations:** a Department of Orthopaedics, Faculty of Medicine, Khon Kaen University, Khon Kaen, Thailand; b Musculoskeletal Oncology Research Group, Khon Kaen University, Khon Kaen, Thailand; c Department of Orthopaedic Surgery, Graduate School of Medical Sciences, Saga University, Saga, Japan; d Department of Orthopaedic Surgery, Graduate School of Medical Sciences, Nagoya city University, Nagoya, Japan.

**Keywords:** case report, L3, local recurrence, spinal metastasis, third lumbar vertebra, total en bloc spondylectomy

## Abstract

**Introduction:**

A posterior-only total en bloc spondylectomy (TES) of the L3 level was deemed a highly intricate surgical procedure, necessitating the preservation of the L3 nerve root to prevent neurological deterioration. Despite bilateral preservation efforts of the L3 nerve roots, neurological deterioration proved unavoidable. This study aims to present the clinical, neurologic, and oncologic outcomes of spinal metastasis patients who underwent a posterior-only approach TES, encompassing the L3 vertebra.

**Materials and methods:**

All patients with L3-involved spinal metastasis undergoing posterior TES between January 2018 and January 2022 were investigated. The primary outcomes considered were the local recurrence rate and manual muscle testing of the lumbar myotome. Secondary outcomes included Frankel neurological status, operative time, blood loss, perioperative and postoperative complications, and Eastern Cooperative Oncology Group score.

**Results:**

Five patients with TES involving L3 (three females) met the inclusion criteria. All patients had solitary metastases (three in the lungs, 2 in the breasts). Postoperatively, all patients experienced weakness of the hip flexors, but they were able to ambulate independently 12 months after surgery. One patient exhibited adjacent segment (L2) disease progression and underwent corpectomy 18 months after TES. No local recurrences at the surgical site were detected on magnetic resonance imaging at the 1-year follow-up.

**Conclusion:**

Posterior-only TES for L3-involved vertebrae yielded excellent results in the local control of metastatic disease. Despite hip flexor weakness, all patients were able to regain independent ambulation after 12 months. TES can offer favorable clinical and oncological outcomes in patients with solitary spinal metastases.

## 1. Introduction

Total en bloc spondylectomy (TES) at the lumbar vertebra is a complex and more operative-related complication than that at the thoracic vertebra, especially if the lesion involves L3, L4, or L5.^[1–8]^ The transection of the L3 to L5 nerve root could lead to deterioration of motor function of the knee extensor or quadriceps and ankle dorsiflexor or tibialis anterior, which impairs the ability to perform basic daily living activities.^[4–7,9]^ However, dissection of the L3 nerve root potentially causes neurapraxia or axonotmesis injury of the nerve and is an obstacle in performing anterior dissection of the vertebra.^[4,6]^

Several studies have reported successful TES for spinal tumors involving the L3 vertebra. Most of the previously published studies were case reports and used various surgical approaches, combined anterior and posterior or single posterior approaches. The aim of the current study was to report the clinical and oncologic outcomes, focusing on local recurrence, in single solitary spinal metastasis patients involving the L3 vertebra who were treated with a single posterior TES with at least 1 year follow up.

## 2. Materials and methods

After the ethical committee of our institution (registration number HE611450) approved the protocol, we prospectively recorded 43 patients with spinal metastasis who underwent TES between January 2018 and January 2022. The inclusion criteria were solitary spinal metastasis involving the L3 vertebra, single posterior, L3 root sparing, TES, and a minimum follow-up time of 12 months. The exclusion criteria were as follows: inability to attend follow-up for at least 12 months and not receiving postoperative magnetic resonance imaging (MRI) at 1 year follow up.

**Figure 1.**
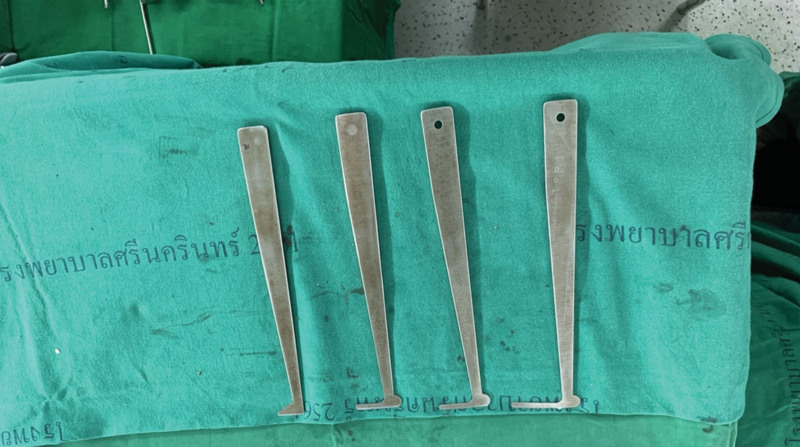
Demonstrated the L-shape chisel that is used for cutting intervertebral disc from posterior to anterior.

All TES procedures were performed by P.P. All patients underwent the posterior approach. En bloc laminectomy was then performed with a posterior approach using a T-saw pediculectomy. En bloc vertebrectomy was performed using a posterior approach by cutting the adjacent intervertebral disc with an L-shaped osteotome (Fig. 1), and then rotating the vertebral body outward carefully to avoid any thecal sac injury.

The primary outcomes were the detection of local recurrence 1 year after TES using MRI and the assessment of manual muscle testing (MMT) of the lumbar myotome immediately post-operative and at every follow-up period. The secondary outcomes were Frankel neurological status, operative time and blood loss, perioperative and postoperative complications, and Eastern Cooperative Oncology Group (ECOG) score. In terms of follow-up, a clinical evaluation was performed, and a plain radiograph was taken monthly for 3 months. A clinical assessment was then performed, and a plain radiograph and computed tomography scan of the spine were performed every 3 months for 1 year. Finally, clinical examination and X-rays were performed every 6 months until year 2. MRI was performed annually to detect local recurrence of metastasis. In terms of neurological status testing evaluation, all patients were assessed by the operating author (P.P.).

## 3. Results

Of the 43 TES cases at our institution, 7 underwent TES at the L3 level. After screening according to the inclusion and exclusion criteria, only 5 patients (3 females) remained in the study. The demographic data of these patients are presented in Table 1.

**Table 1:** Showed patients demographic data.

Patient no	Sex	Age	Histology	Affected vertebra	Pre-operative Frankel grade	Adjuvant treatment	Operative time (min)	Blood loss (L)
1	Female	40	Breast	L3	D	CMT Denosumab	420	4
2	Male	52	Lung	L3	D	CMT + XRT	300	0.8
3	Male	53	Lung	L3	E	CMT + XRT	300	0.3
4	Female	56	Lung	L1-L3	C	CMT + XRT	360	1.5
5	Female	33	Breast	L3	E	CMT + XRT	240	1

CMT = chemotherapy, L = liter, min = minutes, XRT = conventional radiation therapy at surgical sites.

Concerning local recurrence, no instances were detected at the surgical level in the 1-year follow-up MRI. However, 1 patient exhibited disease progression at the adjacent segment (L2) and subsequently underwent corpectomy 18 months after TES.

Postoperatively, all patients had grade 1 or 2 hip flexor weakness on MMT (Table 2). When the Frankel neurological status was calculated, none of the patients showed a postoperative change in status. At 1 year follow up, all patients were able to ambulate independently. The ECOG score remained unchanged or improved after TES. Three of the 5 patients were still attending follow-up for more than 18 months postoperatively. One patient died from diseases that metastasized to other organs, and 1 patient was lost to follow-up 24 months after TES (Table 2).

**Table 2:** Showed the Eastern Cooperative Oncology Group (ECOG), Frankel grading, and manual muscle testing (MMT) of iliopsoas (IP) and quadriceps (Qd) at pre-operative and post-operative follow up until last follow up period.

Patient number	MMT of IP		Last F/U	MMT of Qd		Last F/U	ECOG		Frankel grade		Time to self-ambulation with gait aids	Last follow up time	Current Status
	Pre-op	Post-op		Pre-op	Post-op		Pre-op	Last	Pre-op	Last F/U	(Mo)	(Mo)	
1	4	3	5	4	3	5	3	1	D	E	6	48	Alive
2	4	2	4	4	2	4	3	1	D	D	5	24	Lost F/U
3	5	3	5	5	3	5	2	1	E	E	3	32	Alive
4	4	3	4	4	3	4	3	1	C	E	11	16	Alive
5	5	3	4	5	3	5	3	1	E	E	6	18	Die

The operative time and intra-operative blood loss were shown in Table 2.

Regarding complications, 1 patient experienced superficial wound inflammation that resolved after intravenous antibiotic treatment for 14 days after TES.

## 4. Case presentation

**Figure 2.**
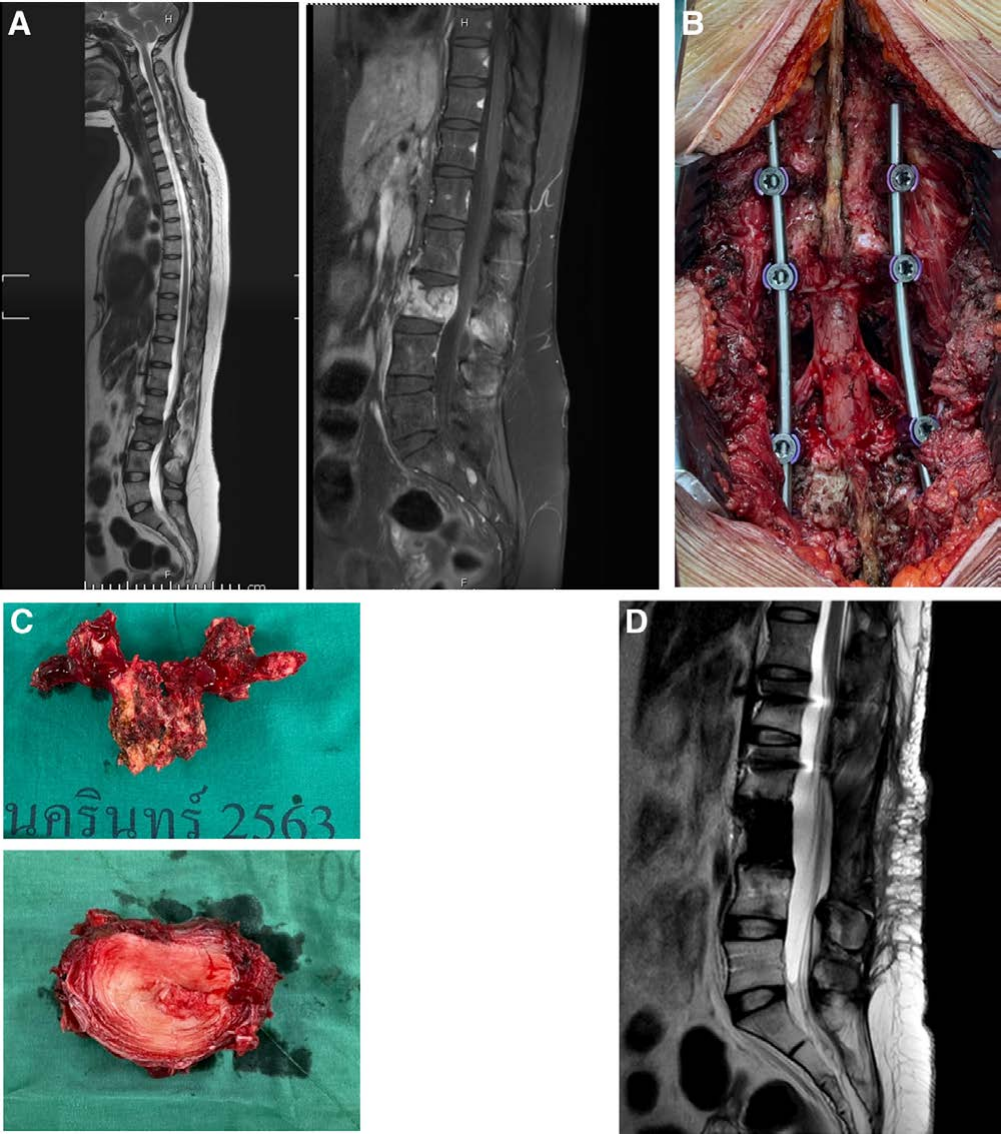
(A) Showed solitary metastasis lesion of L3 in MRI. (B) L3 nerve root sparing through single posterior approach. (C) Showed L3 TES specimen. (D) MRI at 1 yr follow up demonstrated no local recurrence at surgical site. MRI = magnetic resonance imaging, TES = total en bloc spondylectomy.

A 33-year-old female presented with L3 metastasis from breast adenocarcinoma. Whole-spine MRI showed L3 solitary metastasis (Fig. 2A). TES of the L3 was performed using a single posterior approach with L3 nerve root sparing (Fig. 2B). The TES specimen is shown in Figure 2C. At the last follow up period (18 month), the patient could walk independently and showed neurological recovery to Frankel E and an ECOG score of 1. Figure 2D shows no local recurrence at the surgical site on MRI at 1 year follow up.

## 5. Discussion

Solitary spinal metastases can be treated using minimally invasive surgery, separation surgery, debulking or intralesional surgery, and TES. TES is the radical removal of a spinal column-containing tumor.^[10–12]^ The single posterior TES was developed and popularized by Tomita et al^[10–14]^ To facilitate anterior vertebral body dissection and body removal, bilateral nerve roots at the affected vertebral level ligation were recommended. At the lumbar level, especially at L3 to L5, nerve root ligation can cause significant ambulation difficulty.^[1,2,4–6,15]^ However, L3 to L5 nerve root sparing requires careful dissection of the nerve root far enough to create space for anterior body dissection and vertebral body rotation. Despite gentle and meticulous dissection of nerve roots, neurapraxia and axonotmesis can occur postoperatively.

Several authors have reported successful TES at the L3 vertebra using various approaches: the single posterior approach or the combined anterior posterior approach.^[4,6,13]^ Most of these reports were case reports. In our study, we showed that single posterior TES was safe and had a low rate of postoperative complications. Regarding intraoperative blood loss, 1 out of 5 patients suffered from massive blood loss (more than 2 L),^[16]^ which occurred because of difficulty in stopping epidural vein bleeding. No patient demonstrated local recurrence at the resected spinal level, but 1 patient developed a new lesion at the cephalad level (L2) at 18 months, which required an anterior corpectomy procedure. All patients in our study had transient postoperative deterioration of bilateral hip flexors in 1 or 2 grades of MMT. Hip flexor weakness in all patients gradually improved and returned to MMT of at least 4 in all patients at 1 year. Even though hip flexor deterioration occurred after surgery, there was no change in the overall Frankel neurological status of all patients. Regarding wound complications, 1 patient (three levels of TES) developed postoperative wound redness, which resolved after intravenous administration of cefazolin for 14 days. A longer operative time and more soft tissue dissection could be the potential causes of wound complications. For the overall performance, all 5 patients showed the same or improved ECOG scores after TES.

The limitations of our study were as follows: the relatively small number of patients and the absence of a comparison group with other types of surgical treatments, such as palliative or separation surgery. Our study had the following strengths: the inclusion of only a single posterior TES at L3 and MRI imaging at the 1-year follow-up to detect local recurrences.

In conclusion, a single posterior TES at the spinal level involving L3 was effective for the local control of metastasis. Despite immediate postoperative neurological deterioration of the hip flexor, the function gradually improves over time within 1 year. TES could be considered a surgical option in patients with solitary spinal metastasis, especially in those who need local control of the disease.

## Acknowledgments

We thank the patients and the Department of Orthopedics and Faculty of Medicine for their support.

## Author contributions

**Conceptualization:** Permsak Paholpak, Tadatsugu Morimoto, Winai Sirichativapee, Wilasinee Sirichativapee, Yuichi Kasai, Hideki Murakami.

**Data curation:** Permsak Paholpak.

**Formal analysis:** Permsak Paholpak.

**Funding acquisition:** Permsak Paholpak.

**Investigation:** Permsak Paholpak.

**Methodology:** Permsak Paholpak.

**Project administration:** Permsak Paholpak.

**Resources:** Permsak Paholpak.

**Software:** Permsak Paholpak.

**Supervision:** Permsak Paholpak, Taweechok Wisanuyotin, Winai Sirichativapee.

**Validation:** Permsak Paholpak.

**Visualization:** Permsak Paholpak.

**Writing – original draft:** Permsak Paholpak.

**Writing – review & editing:** Permsak Paholpak, Tadatsugu Morimoto, Taweechok Wisanuyotin, Winai Sirichativapee, Wilasinee Sirichativapee, Weerachai Kosuwon, Yuichi Kasai, Hideki Murakami.
